# Type I and Type II Interferon Coordinately Regulate Suppressive Dendritic Cell Fate and Function during Viral Persistence

**DOI:** 10.1371/journal.ppat.1005356

**Published:** 2016-01-25

**Authors:** Cameron R. Cunningham, Ameya Champhekar, Michael V. Tullius, Barbara Jane Dillon, Anjie Zhen, Justin Rafael de la Fuente, Jonathan Herskovitz, Heidi Elsaesser, Laura M. Snell, Elizabeth B. Wilson, Juan Carlos de la Torre, Scott G. Kitchen, Marcus A. Horwitz, Steven J. Bensinger, Stephen T. Smale, David G. Brooks

**Affiliations:** 1 Department of Microbiology, Immunology, and Molecular Genetics, University of California, Los Angeles, Los Angeles, California, United States of America; 2 Department of Medicine, David Geffen School of Medicine, University of California, Los Angeles, Los Angeles, California, United States of America; 3 Division of Hematology and Oncology, Department of Medicine, UCLA AIDS Institute and the Eli and Edythe Broad Center of Regenerative Medicine and Stem Cell Research, David Geffen School of Medicine, University of California, Los Angeles, Los Angeles, California, United States of America; 4 Princess Margaret Cancer Center, Immune Therapy Program, University Health Network, Toronto, Ontario; 5 Department of Immunology and Microbial Science, The Scripps Research Institute, La Jolla, California, United States of America; 6 Department of Molecular and Medical Pharmacology, David Geffen School of Medicine, University of California, Los Angeles, Los Angeles, California, United States of America; 7 Department of Immunology, University of Toronto, Toronto, Ontario, Canada; New York University, UNITED STATES

## Abstract

Persistent viral infections are simultaneously associated with chronic inflammation and highly potent immunosuppressive programs mediated by IL-10 and PDL1 that attenuate antiviral T cell responses. Inhibiting these suppressive signals enhances T cell function to control persistent infection; yet, the underlying signals and mechanisms that program immunosuppressive cell fates and functions are not well understood. Herein, we use lymphocytic choriomeningitis virus infection (LCMV) to demonstrate that the induction and functional programming of immunosuppressive dendritic cells (DCs) during viral persistence are separable mechanisms programmed by factors primarily considered pro-inflammatory. IFNγ first induces the *de novo* development of naive monocytes into DCs with immunosuppressive potential. Type I interferon (IFN-I) then directly targets these newly generated DCs to program their potent T cell immunosuppressive functions while simultaneously inhibiting conventional DCs with T cell stimulating capacity. These mechanisms of monocyte conversion are constant throughout persistent infection, establishing a system to continuously interpret and shape the immunologic environment. MyD88 signaling was required for the differentiation of suppressive DCs, whereas inhibition of stimulatory DCs was dependent on MAVS signaling, demonstrating a bifurcation in the pathogen recognition pathways that promote distinct elements of IFN-I mediated immunosuppression. Further, a similar suppressive DC origin and differentiation was also observed in *Mycobacterium tuberculosis* infection, HIV infection and cancer. Ultimately, targeting the underlying mechanisms that induce immunosuppression could simultaneously prevent multiple suppressive signals to further restore T cell function and control persistent infections.

## Author Summary

Persistent virus infections induce host derived immunosuppressive factors that attenuate the immune response and prevent control of infection. Although the mechanisms of T cell exhaustion are being defined, we know surprisingly little about the underlying mechanisms that induce the immunosuppressive state and the origin and functional programming of the cells that deliver these signals to the T cells. We recently demonstrated that type I interferon (IFN-I) signaling was responsible for many of the immune dysfunctions associated with persistent virus infection and in particular the induced expression of the suppressive factors IL-10 and PDL1 by dendritic cells (DCs). Yet, mechanistically how IFN-I signaling specifically generates and programs cells to become immunosuppressive is still unknown. Herein, we define the underlying mechanisms of IFN-I mediated immunosuppression and establish that the induction of factors and the generation of the DCs that express them are separable events integrally reliant on additional inflammatory factors. Further, we demonstrate a similar derivation of the suppressive DCs that emerge in other diseases associated with prolonged inflammation and immunosuppression, specifically in HIV infection, *Mycobacterium tuberculosis*, and cancer, indicating a conserved origin of immunosuppression and suggesting that targeting the pathways that underlie expression of immunosuppressive cells and factors could be beneficial to treat multiple chronic diseases.

## Introduction

Unlike immune responses against most infections, the response against persisting viruses rapidly becomes dysfunctional and unable to purge infection. The prolonged virus replication progressively induces a deterioration of the T cell response, a phenomenon termed exhaustion [1]. T cell exhaustion involves a specific molecular and metabolic program, functionally characterized by altered cytokine production in conjunction with decreased proliferative capacity and cellular cytotoxicity. Importantly, the restoration of exhausted T cell functions can lead to the control of a persistent virus infection [2–4], suggesting that overcoming immunosuppression is a potent path to control persistent virus infections. Similar parameters of immune dysfunction are observed during many persistently viremic infections including, HIV, HBV and HCV infection in humans, SIV in monkeys and lymphocytic choriomeningitis virus (LCMV) infection in mice, indicating that persisting viral replication initiates a conserved immune differentiation program across different species of pathogen and host [5]. Thus, understanding the mechanisms that drive immunosuppression and how to overcome them will be critical to restore and then maintain immune-mediated control of persistent virus infections.

Many of the suppressive factors that negatively regulate the T cell response are being identified and their modulation is revolutionizing medicine. However, we still know surprisingly little about the mechanisms that induce, regulate and sustain the expression of the suppressive factors themselves and the cell types that express them in chronic disease. In response to viral persistence, the host initiates an immunosuppressive program through factors such as interleukin-10 (IL-10) and programmed cell death ligand 1 (PDL1) that actively suppress antiviral T cell responses and facilitate persistent infection [1–4]. Importantly, we demonstrated that many cell types are capable of producing IL-10 and PDL1 during persistent infection and it is likely that all these work in combination and in their specific niches to establish the overall suppressive environment and inhibition of the antiviral response [6]. In an effort to understand how the suppressive factors are regulated, we identified distinct populations of immunoregulatory (ireg) antigen presenting cells (APCs), including dendritic cells (DC) and macrophages that simultaneously express multiple inhibitory factors to suppress T cell responses (e.g. IL-10, PDL1, PDL2, indoleamine 2,3 dioxygenase, IDO) [6]. A different population of macrophages was recently identified in persistent LCMV infection resembling the myeloid derived suppressor cell (MDSC) observed in cancer [7]; however, these MDSC-like cells were distinct from DC and macrophages we identified and did not express IL-10 or PDL-1. Considering the fundamental role of IL-10 and PDL1 in suppressing T cells and limiting immune mediated control of persistent virus infection, the concentration of these factors onto specific iregAPC subsets indicates a mechanism to deliver multiple potent inhibitory signals to T cells in a single interaction; and as such, these iregAPC represent a centralized node of immunosuppressive signals.

Counter to immunosuppression, persistent virus infections are also characterized by chronic production of pro-inflammatory factors that associated with worsened disease progression [8, 9]. Yet, how these two seemingly opposed pro- and anti-inflammatory programs co-exist and regulate each other is not well understood. We recently established a link between chronic type I interferon (IFN-I) signaling, immune suppression and viral persistence [10]. Simultaneous with its critical antiviral role, IFN-I signaling led to a surprising amount of the immune dysfunctions associated with viral persistence including expression of IL-10 and PDL1 [10, 11]. Antibody blockade of the IFN-I receptor (IFNR) during persistent LCMV infection diminished the expression of IL-10 and PDL1, reversed many of the immune defects, and ultimately facilitated long-term virus control [10–12]. Thus, in addition to its antiviral and immune stimulatory roles, IFN-I signaling also regulates multiple suppressive pathways and dysfunctions that facilitate viral persistence. Interestingly, many of the immune dysfunctions restored by blocking IFN-I signaling are also linked to IL-10 and PDL1, yet aside from the association, a mechanistic understanding of how IFN-I promotes IL-10 and PDL1 expression is lacking. In particular, whether IFN-I specifically leads to the genesis of cells that as part of their program produce IL-10 and PDL1 or whether IFN-I acts upon existing cells to endow them with suppressive activity. Herein we demonstrate that the *de novo* generation of DC with T cell suppressive potential and the induction of their immunosuppressive program is a collaboration between the interferon systems. First, IFNγ is required to drive monocytes to differentiate into DC with suppressive potential and second; IFN-I targets these DC to directly induce the immunosuppressive factors IL-10 and PDL1. In parallel to induction of suppressive factors, IFN-I inhibits the emergence of DC with T cell stimulatory capacity, in essence shaping the immunosuppressive environment. Chronic IFN-I signaling, suppressive APC, immunosuppression and impaired T cell responses are not limited to persistent virus infections, but are also observed in other chronic diseases, including bacterial infections (e.g., *Mycobacterium tuberculosis*; *Mtb*) and cancer [5, 13–15]. We further demonstrate the emergence and monocyte-origin of iregDC in cancer, as well as *Mtb* and HIV infection, implicating their common origin and differentiation in diverse situations of chronic disease.

## Results

### Anatomical localization of IL-10 expressing cells *in vivo*

**Fig 1:**
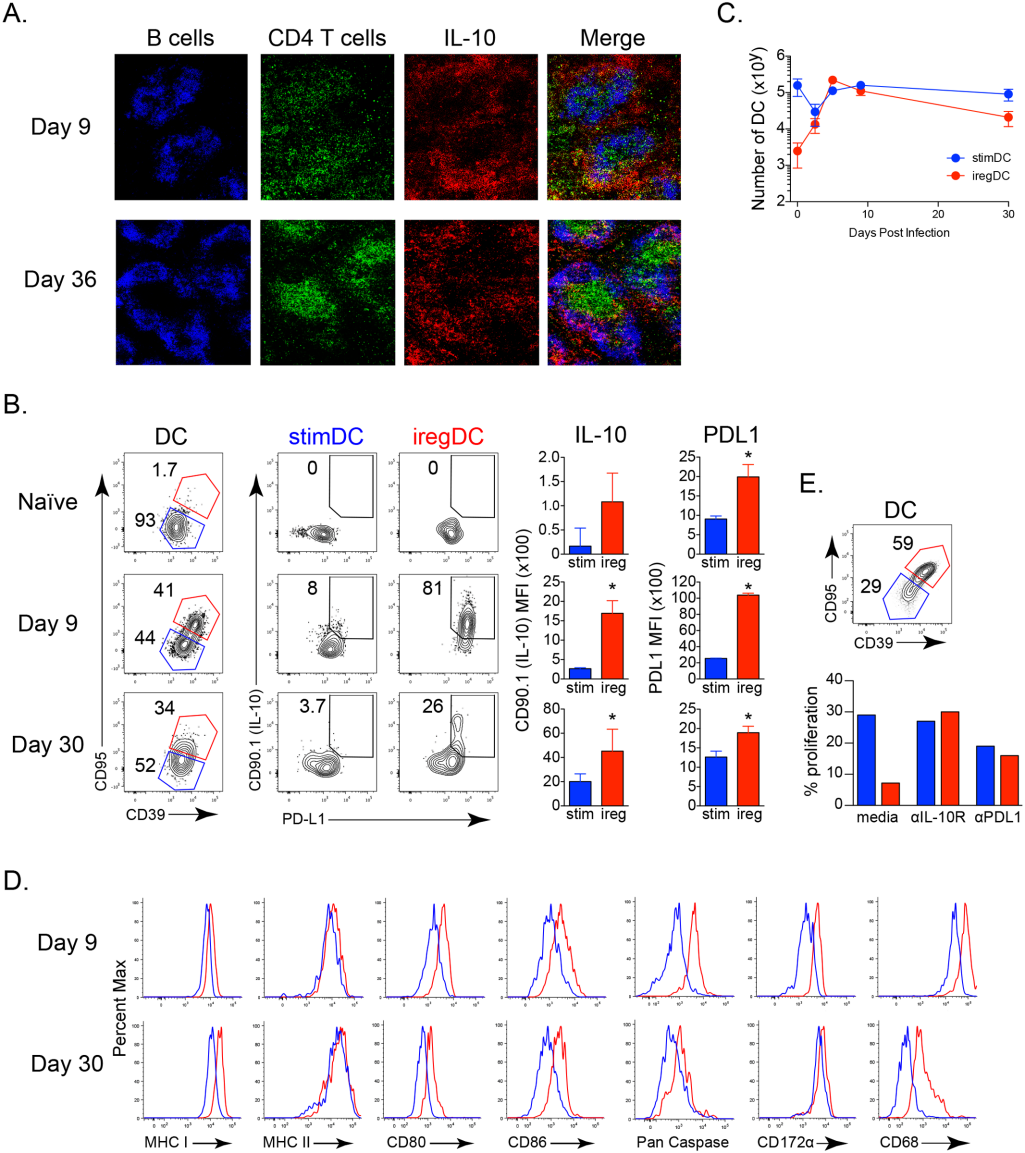
*In vivo* localization and identification of immunoregulatory DCs during viral persistence. **A.** Sections from IL-10 reporter mice infected with LCMV-Cl13 for 9 or 36 days were stained for B cells, CD4+ T cells, and CD90.1 (IL-10) and visualized at 10x magnification. **B.** CD39 and CD95 expression on splenic CD11b+ DCs from naive IL-10 reporter mice or IL-10 reporter mice infected with LCMV-Cl13 for 9 or 30 days and their corresponding expression of CD90.1 (IL-10) and PDL1 within the iregDC (red) and stimDC (blue) populations. Bar graphs indicate the geometric mean fluorescence intensity (MFI) of CD90.1 (IL-10) and PDL1 expression by iregDCs (red) and stimDCs (blue). DC are characterized as being viability dye-, CD45+, Thy1.2-, NK1.1-, Ly6G-, CD11c++ (high), CD11b+. **C.** The number of iregDCs (red) and stimDCs (blue) based on CD39 and CD95 expression at the indicated time point after LCMV-Cl13 infection. **D.** Histograms of the indicated protein on iregDCs (red) and stimDCs (blue) at day 9 and day 30 following LCMV-Cl13 infection. **E.** iregDCs and stimDCs were sorted from splenocytes at day 9 of LCMV-Cl13 infection and cultured with LCMV specific CD4+ T cells (SMARTA) for 3 days with IL-10R blocking antibody, PDL1 blocking antibody, or media alone. Bar graph represents the proportion of proliferated SMARTA cells after the culture. Data in 1E show a single experiment using iregDC and stimDC sorted from a pool of 8 mice in order to obtain adequate numbers of each population. Data are representative of 2 or more independent experiments each consisting of 3–4 mice per group. *, p<0.05.

To explore the mechanisms underlying the generation and potentiation of immunosuppression during persistent virus infection, we used the LCMV model. Infection with the LCMV variant Clone 13 (Cl13) establishes a persistent infection due to increases in virus replication and receptor affinity that help to outcompete the developing immune response, thereby inducing immunosuppression and T cell exhaustion [16, 17]. We first sought to determine the anatomical localization of IL-10 expressing cells *in vivo* and whether they localize to defined foci or are distributed throughout the tissue. Using IL-10 reporter mice[18], we observed that at day 9 after LCMV-Cl13 infection, IL-10 expressing cells were distributed throughout the red pulp and marginal zone of the spleen consistent with DC and macrophage localization of IL-10 at this time point during infection (Fig 1A and S1A Fig) [6]. As infection progressed, the amount of IL-10 expressing cells decreased[6], but they were still largely observed in the red pulp and marginal zone, although there was also dispersal to other areas by this time (Fig 1A). Thus, as opposed to localized defined foci, IL-10 expressing cells are dispersed throughout the spleen and essentially form a ‘blanket’ throughout the APC: T cell area during persistent virus infection.

### Immunosuppressive iregAPC express distinct molecular and cellular profiles

We have previously utilized IL-10 reporter mice to identify the iregAPC [6], however this limits the ability to differentiate factors affecting their generation from functional changes or to elucidate the mechanisms underlying their suppressive capacity using other mouse and infection models. To overcome the reliance on IL-10 reporter mice, we identified a panel of markers increased on IL-10/PDL1 co-producing iregDC (Fig 1B and 1C, S1B and S1C Fig). Compared to their stimDC counterparts, iregDC could be identified by their increased expression of CD95 (Fas) and CD39 (an ectoenzyme responsible for deactivating extracellular ATP [19]) as well as high levels of pan-Caspase activity (including Casp1, but also other Caspases since high pan-Caspase activity is still observed in Caspase1-/- mice) (Fig 1B and 1D). Further, factors associated with phagocytosis and degradation such as CD172α and CD68, as well as CD73 that works in conjunction with CD39 to convert ATP to adenosine, were also increased on iregDC (Fig 1D). CD39+/CD95+ iregDCs expressed high levels of MHC and CD80/86 to interact with T cells, but unlike CD39-/CD95- stimDC that readily activated T cells and were not affected by anti-IL-10R or anti-PDL1 blockade, iregDC suppressed T cell activation in an IL-10 and PDL1 dependent manner (Fig 1D and 1E). At day 30 after LCMV-Cl13 infection, the amount of IL-10 expressing iregDC decreased, but those that remained were still identified based on CD39 and CD95 expression (Fig 1B and 1C). Importantly, these markers could also be used to identify iregDC in LCMV-Cl13 infected Balb/c mice (S1D Fig), indicating that iregDC development is not C57BL/6 strain specific and establishing a panel of markers to distinguish suppressive DC without reporter mice.

**Fig 2:**
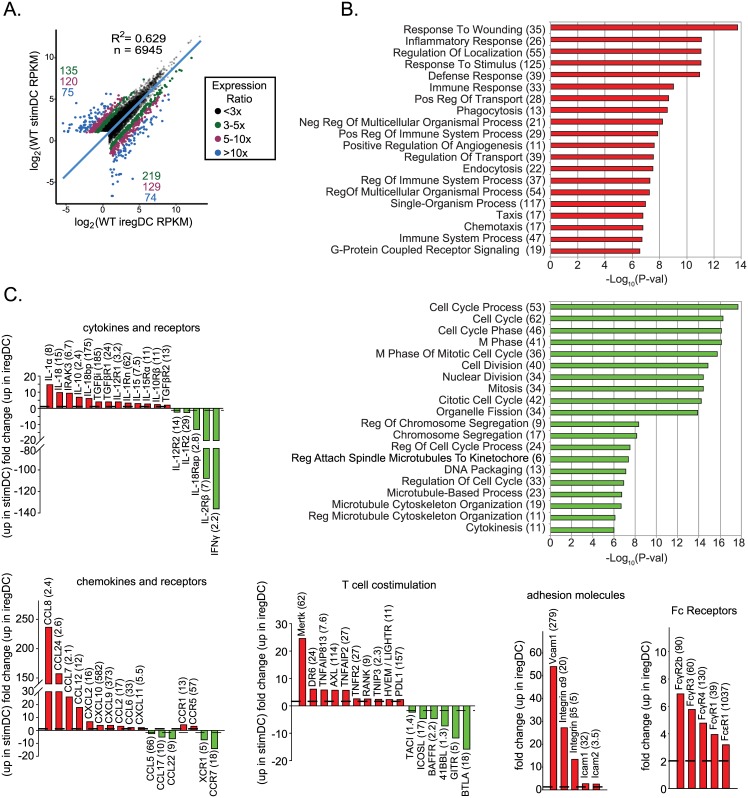
iregDCs have a distinct expression profile compared to stimDCs. StimDCs and iregDCs were obtained from day 9 LCMV-Cl13 infected mouse splenocytes, RNA was harvested and subjected to RNA-seq. **A.** Graph scatter shows the log-transformed expression values for genes expressed at 2 RPKM or more in at least one sample. Dots are color-coded based on the ratio of RPKM values to indicate differentially expressed (DE) genes between the two cell types. The number of genes in each DE group are indicated in the top left (higher in stimDCs) and bottom right (higher in iregDCs) corners. Selected DE genes from this dataset are shown in the rest of the panels to highlight important gene groups as indicated on top of each panel. **B.** Gene Ontology (GO) term over representation analysis was performed on a set of genes that were at least 5-fold differentially expressed between the iregDC and stimDC samples. Plots show the statistical significance of GO terms that are enriched in genes expressed at higher levels in iregDCs and stimDCs. The number of genes that were classified under a particular GO term are indicated in brackets. GO terms with an adjusted (Benjamini-Hochberg method) p-value of < = 0.05 were considered to be enriched in the DE gene set. **C.** Bar graphs show the fold change in the indicated gene. Up on the y-axis indicates increase in iregDC (red). Down on the y-axis indicates increase in stimDC (green). The number in parenthesis indicates the RPKM value of the highest sample (to indicate relative expression level).

To profile iregDC vs. stimDC molecularly, we performed RNA-seq analysis 9 days after persistent LCMV-Cl13 infection. Expression analysis indicated a large overlap between the two populations, with approximately 750 genes differentially expressed by 3-fold or more (assuming an RPKM cutoff of 2 in at least one of the samples) (Fig 2A). Interestingly ~150 genes were differentially expressed greater than 10-fold (Fig 2A). Gene ontology analysis indicated iregDC increased expression of genes involved in inflammatory responses, wound healing and phagocytosis/Fc receptor expression, while stimDCs were enriched for multiple pathways involved in cell cycle progression (Fig 2B and 2C). These analyses further identified differential gene expression of key immunomodulatory cytokines, chemokines and T cell costimulatory molecules (Fig 2B and 2C). iregDCs exhibited increased expression of T cell attracting chemokines [CXCL9 (RPKM 373) and CXCL10 (RPKM 582)] and monocyte attracting chemokines (CCL7, CCL8 and CCL12) which bind to CCR1, CCR2 and CCR5 (upregulated on iregDCs) and could serve to further attract iregDC to sites of inflammation (Fig 2C). Although expression of the costimulatory CD80 and CD86 proteins was similar on iregDCs and stimDCs (Fig 1D), iregDC expressed decreased levels of multiple T cell costimulatory molecules that positively activate, modulate and sustain antiviral and antitumor T cell functions, including ICOSL, 4-1BBL, GITR and TACI and increased anti-inflammatory receptors, including Mertk and Axl (Fig 2C) [16, 20]. Consistent with increased inflammasome [10] and Caspase activity (Fig 1D), iregDC have increased expression of IL-1a and IL-18 RNA which could further contribute to ongoing chronic immune activation during viral persistence (Fig 2C). On the other hand, iregDC expressed low levels of IFNγ (which is antiviral and helps drive Th1 responses), but instead expressed elevated IL-10 and PDL1 to further blunt Th1 immunity and suppress T cell responses (Fig 2C). Thus, iregDCs express increased levels of genes involved in recruiting T cells, but upon interaction have decreased levels of stimulatory proteins and increased inhibitory factors to blunt T cell responses.

### Direct IFN-I signaling drives immunosuppressive function and inhibits stimulatory DC generation, but does not underlie iregDC fate determination

**Fig 3:**
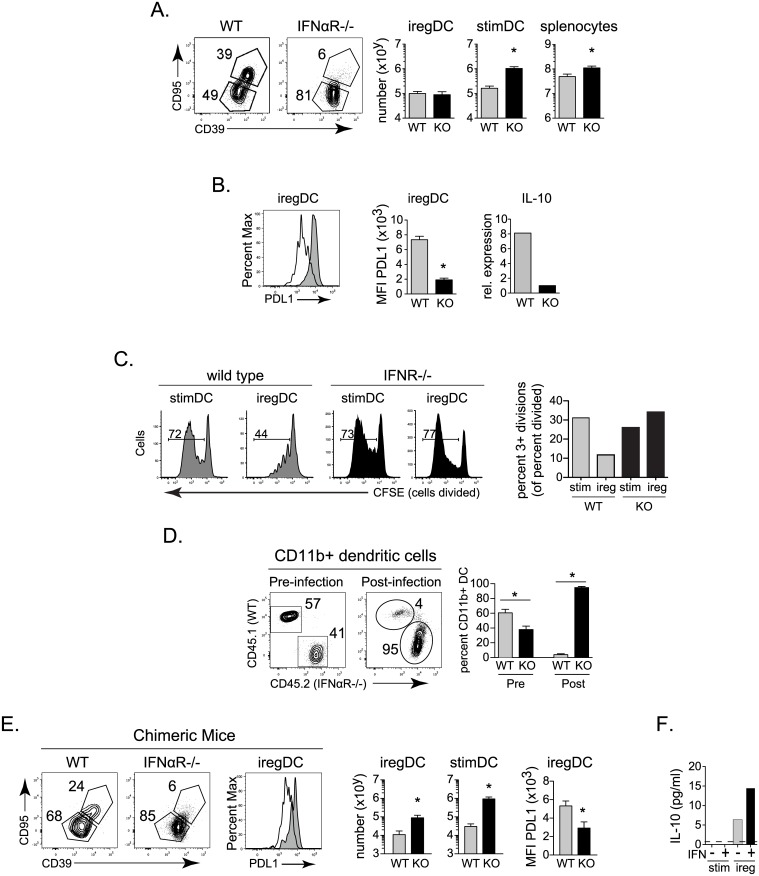
Two-pronged mechanism of IFN-I mediated immunosuppression: IFN-I programs iregDC suppressive functions and limits stimDC generation. **A.** CD39 and CD95 expression by splenic CD11b+ DCs from WT and IFNαR-/- mice at day 9 after LCMV-Cl13 infection. Bar graphs indicate the number of iregDC (CD39+, CD95+), stimDC, and total splenocytes. **B.** Histogram shows PDL1 expression on iregDCs from WT (grey) and IFNαR-/- (white) mice. Bar graphs show quantification of PDL1 expression (MFI) and IL-10 RNA by iregDC from WT and IFNαR-/- mice at day 9 of LCMV-Cl13 infection. To obtain sufficient cell numbers for the IL-10 RNA analysis, iregDC and stimDC were sorted from a pool of 8 mice. Data for IL-10 RNA analysis show one of two experiments. **C.** StimDC and iregDC were FACSorted from WT or IFNR-/- mice 9 days after LCMV-Cl13 infection and co-cultured with CFSE labeled naïve LCMV-specific CD4 SMARTA T cells. No peptide was added to the cultures. Flow plots show CFSE dilution by the virus-specific CD4 T cells 5 days after co-culture. Numbers in the flow plot indicate the percent of virus-specific CD4 T cells that have diluted CFSE. The bar graph indicates the percent of virus-specific CD4 T cells that have undergone 3 or more divisions (gated on divided cells), calculated as in [21]. **D.** Flow plots show reconstitution of CD11b+ DCs from WT CD45.1+) and IFNαR-/- CD45.2+) lineage cells in bone marrow chimera pre-infection (blood– 9 weeks post reconstitution) and post-infection (spleen—day 9 of LCMV-Cl13 infection). Bar graph shows quantification of the percent of lineage derived CD11b+ DCs. **E.** Flow plots show iregDC and stimDC gated on splenic CD11b+ DCs at day 9 after LCMV-Cl13 infection of the WT: IFNαR-/- bone marrow chimera mice. Flow plots are gated on the WT and IFNαR-/- lineage from the same mouse. Histogram shows PDL1 expression on iregDCs from WT (grey) and IFNαR-/- (white) lineage cells in the same chimeric mice. Bar graph indicates the total number of iregDC and stimDC and PDL1 MFI from each lineage in the chimeric mice. **F.** Bar graph shows IL-10 expression in the supernatant of sorted CD11b+ DC after stimulation with media alone or IFNβ. Supernatants were harvested 24 hours after culture. Data are representative of 2 or more independent experiments each consisting of 3–4 mice per group. *, p<0.05. For the WT vs IFNR-/- DC co-culture with T cells (Fig 3C), data are shown from 1 of 2 experiments each using samples pooled from 10–15 mice per DC group to obtain adequate numbers of each DC population.

How IFN-I triggers the expression of IL-10 and PDL1 remains poorly understood. IFN-I could directly signal pre-existing iregDC to induce IL-10 and PDL1 expression or IFN-I could induce the differentiation of iregDC, which as part of their inherent programming, are immunosuppressive. To distinguish between these mechanisms, we utilized the iregDC markers CD39 and CD95. The frequency of iregDC was severely diminished in LCMV-Cl13 infected IFNαR-/- mice compared to wild type (WT) mice (Fig 3A). However, the number of iregDC was unchanged between WT and IFNαR-/- mice (Fig 3A), while the number of IFNαR-/- stimDC were increased ~6-fold compared to WT mice (Fig 3A). Together, these data suggest that iregDC generation still occurs in the absence of IFN-I signaling, whereas IFN-I suppresses the expansion of the stimDC population.

We next determined the role of IFN-I signaling toward expression of the suppressive factors IL-10 and PDL1 by the iregDC and their ability to suppress antiviral T cell activation. Whereas WT iregDC expressed high levels of PDL1 and IL-10, IFNαR-/- iregDC (although present at the same amount) exhibited substantially decreased expression of PDL1 and IL-10 compared to WT mice (Fig 3B), suggesting that although the generation of the cells with suppressive potential was not dependent on IFN-I signaling, IFN-I signaling is required for expression of their suppressive program. Interestingly, ireg phenotype DC that could not receive IFN-I signals lost their suppressive activity and now stimulated robust antiviral T cell responses similar to WT and IFNαR-/- stimDC (Fig 3C). In addition to a decrease in the overall amount of cells that divided, the substantial blockade of later cell divisions by WT iregDC (i.e., cells undergoing 3+ divisions) was not observed in IFNαR-/- iregDC (Fig 3C). CFSE dilution was calculated as described by Gett et al [21] to accurately quantify the amount of divided cells. Thus, the expression of IL-10 and PDL1 by iregDC and their ability to subsequently suppress antiviral T cell responses is dependent on IFN-I signaling and in the absence of IFN-I, these otherwise suppressive DC now stimulate robust T cell responses.

To determine whether intrinsic IFN-I signaling was required for inducing the suppressive function by iregDC, we utilized 50:50 WT: IFNαR-/- bone marrow chimera mice wherein WT and IFNαR-/- cells are present within the same environment and exposed to the same signals. Interestingly, prior to infection, all subsets of WT cells analyzed except B cells were increased (Fig 3D and S2A Fig), indicating that in competition, cells that cannot receive steady state IFN-I signals are hindered. Conversely, following LCMV-Cl13 infection, IFNαR-/- macrophages and particularly DCs were now present at much higher levels compared to their WT counterparts (Fig 3D and S2A Fig). Interestingly, IFNαR-/- CD4 T cells and B cells, two populations we have previously shown to be increased in IFNαR-/- mice and anti- IFNαR antibody treated mice [10], were not increased above their pre-infection levels following persistent infection of mixed bone marrow chimera mice (S2A Fig), indicating that their expansion in IFNαR-/- mice and anti- IFNαR antibody treated mice is not cell intrinsic. Similar to IFNαR-/- mice, the frequency of iregDC in mixed chimeras from the IFNαR-/- lineage was greatly reduced compared to WT cells (Fig 3E). However, due to the advantage that IFNαR-/- DCs had compared to the WT DCs, there were numerically more IFNαR-/- iregDCs (Fig 3E), again consistent with IFN-I being dispensable for iregDC generation. Further, the number of IFNαR-/- stimDCs were increased, indicating that the suppression of stimDCs is similarly directly mediated by IFNαR intrinsic signals. On the other hand, PDL1 expression was decreased on the IFNαR-/- iregDC in the mixed chimera mice and IFN-I treatment significantly upregulated IL-10 expression by DC (Fig 3E and 3F), indicating that IFNαR signaling directly induced suppressive factors on pre-existing cells. Thus, the immunosuppressive impact of IFN-I signaling is two pronged: IFN-I signaling limits the expansion of the stimDCs population while endowing the iregDC population with the capacity to generate immunosuppressive factors and repress antiviral T cell stimulation.

### iregDC and stimDC are ontologically distinct

**Fig 4:**
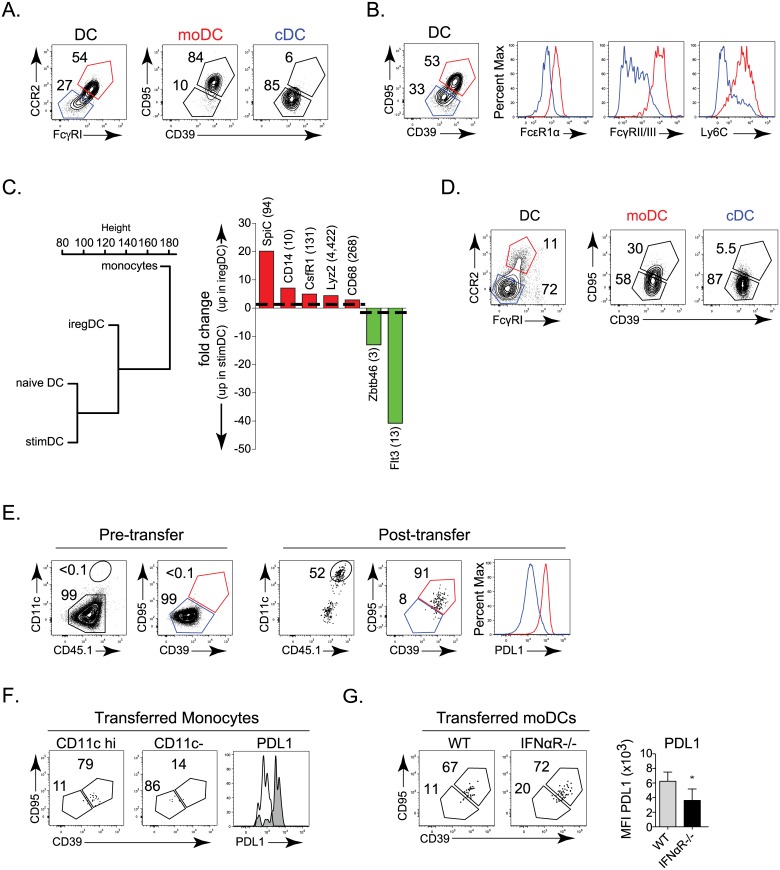
Suppressive iregDC are of monocyte origin. **A.** Flow plots show CCR2 and FcγRI expression on splenic CD11b+ DCs on day 9 after LCMV-Cl13 infection. CCR2+, FcγRI+ (red) and CCR2-, FcγRI- (blue) populations are subsequently plotted on iregDC markers CD95 and CD39. **B.** Expression of FcεR1α, FcγRII/III, and Ly6C on iregDCs (red) and stimDCs (blue) gated on CD95 and CD39 from CD11b+ DCs on day 9 after LCMV-Cl13 infection. **C.** Dendogram representing the gene expression profile relation between naïve monocytes, naïve DCs, iregDCs and stimDCs at day 9 of LCMV-Cl13 infection. Bar graph of the expression of key monocyte-associated (red) and conventional DC-associated (green) genes. Numbers in parenthesis indicate the RPKM value for the highest sample. **D.** Left flow plots depict CCR2 and FcγRI expression on splenic CD11b+ DCs at day 25 after LCMV-Cl13 infection. Right flow plots depict CD39 and CD95 expression gated on CCR2+, FcγRI+ (red) and CCR2-, FcγRI- (blue) populations. **E.** Monocytes were isolated from naïve WT CD45.1+ mice and transferred into WT CD45.2+ mice that had been infected for 3 days with LCMV-Cl13. Left flow plots show DC and iregDC marker expression on isolated monocytes prior to transfer (gated on CCR2+, Ly6C+, FcγRI+ cells). Right flow plots depict transferred monocytes on day 6 post-transfer (day 9 after infection; gated on CD45.1+, CCR2 +, FcγRI+, CD11b+ cells). Histogram represents the expression of PDL1 of the transferred monocytes based on iregDC (red) and stimDC (blue) gates. **F.** Naïve CD45.1+ monocytes were transferred into WT CD45.2+ mice that had been infected with LCMV-Cl13 for 19 days. Six days later (day 25 after infection), DC and iregDC differentiation were analyzed in the spleen. Flow plots are gated on the transferred CD45.1+, CCR2+, FcγRI+ cells and then into the CD11c high (DC) and CD11c- populations. The histogram shows the expression of PDL1 on the CD11c high (shaded grey) and CD11c- (solid black line) monocyte derived subsets 6 days after transfer. **G.** Naïve WT or IFNαR-/- monocytes were transferred into LCMV-Cl13 infected WT mice as in E. Flow plots show CD39 and CD95 expression on transferred monocytes in the spleen on day 9 after LCMV-Cl13 infection (gated on CCR2+, FcγRI+, CD11c++, CD11b+ DC). Bar graph indicates PDL1 expression on iregDCs derived from the transferred monocytes. Data are representative of 2 or more independent experiments each consisting of 3–4 mice per group. *, p<0.05. RNA-seq is from 1 experiment using samples each pooled from 10 mice per group.

Since IFN-I signaling induces the suppressive program that inhibits T cell activation, but not the choice to become iregDC, an unanswered question is why do some CD11b+ DCs become iregDCs while others become stimDCs since IL-10 expressing cells are dispersed throughout the tissue and therefore are all present in the same environment? To understand the origin of iregDC and whether they are *de novo* derived or transformed from a previously existing DC population, we analyzed different DC subsets. At day 9 after LCMV-Cl13 infection, iregDC expressed increased levels of the monocyte-associated proteins CCR2 (C-C chemokine receptor type 2) and FcγRI (CD64) in conjunction with the other monocyte-associated proteins FcεR1α, FcγRII/III, and Ly6C (Fig 4A and 4B). RNA-seq global transcriptional profiles further placed iregDC as an intermediate population between naïve monocytes and DC (Fig 4C), suggesting monocyte differentiation into DC-like cells. Conversely, stimDCs exhibited a gene ontogeny profile similar to naïve DC and expressed higher levels of the DC associated marker FLT3 and transcription factor Zbtb46 (Fig 4C). As persistent infection progressed, the CD39+ and CD95+ iregDC continued to express the monocyte-associated proteins CCR2 and FcγRI, although at this point many monocyte-derived DC were now not iregDC (Fig 4D), consistent with the decreased IL-10 and PDL1 at this time point (Fig 1). Thus, a bifurcation exists during viral persistence wherein monocytes can give rise to suppressive iregDC whereas stimDCs are conventional DCs (cDCs).

To further interrogate the ability of monocytes to develop into iregDC in the context of persistent virus infection, we transferred monocytes from naïve mice into persistently infected mice. Directly after isolation naïve monocytes are CCR2+, FcγRI+ and Ly6C+, but do not express appreciable levels of CD11c or other iregDC markers (Fig 4E and S2B Fig). Similarly, transfer of naïve monocytes into naïve mice did not induce iregDC marker expression or PDL1 upregulation, although a fraction of the monocytes did become CD11c high, suggesting that iregDC differentiation requires infection/inflammation. Following transfer into LCMV-Cl13 infected mice, a substantial population of naïve monocytes upregulated CD11c and differentiated into iregDCs (Fig 4E). Further, naïve monocytes transferred 19 days after LCMV-Cl13 infection (i.e., an established persistent infection) continued to develop into iregDC (Fig 4F), indicating that *de novo* monocyte into iregDC differentiation is sustained throughout persistent virus infection. When IFNαR-/- naïve monocytes were transferred, CD39+, CD95+ iregDC development was observed, but the cells expressed decreased PDL1 (Fig 4G), further substantiating that IFN-I is not required for iregDC generation, but endows the cells with suppressive activity.

### Cell extrinsic MyD88 and direct IFNγ signaling, but not direct virus infection, are required for iregDC generation

**Fig 5:**
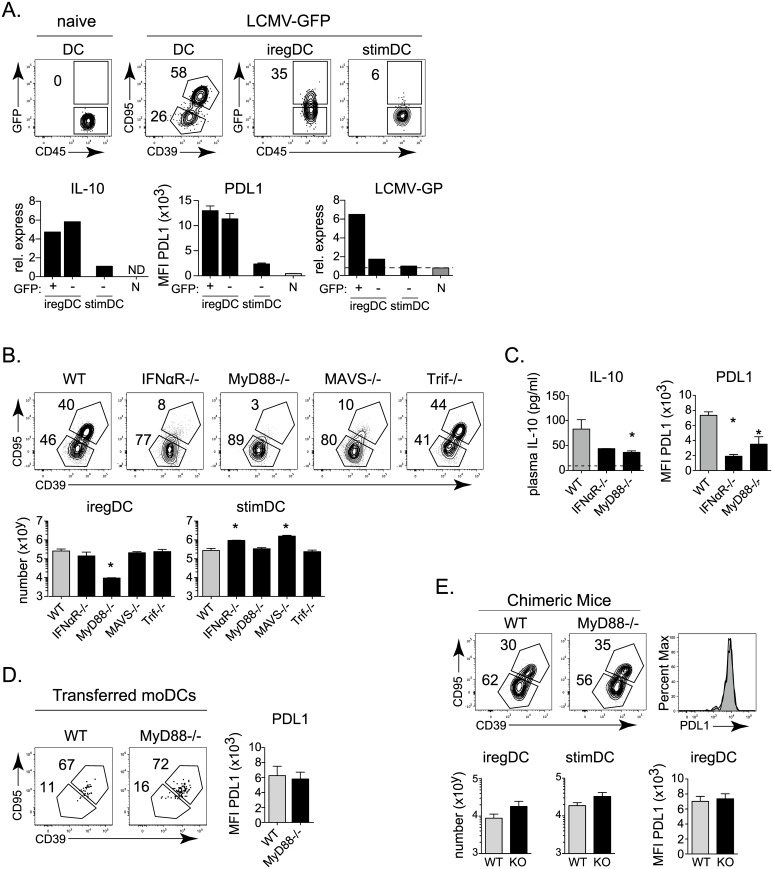
iregDC extrinsic MyD88 signaling, but not direct LCMV infection or IFNβ signaling is required for iregDC differentiation. **A.** WT mice were CD8 depleted and then infected with LCMV-Cl13-GFP. Flow plots show GFP expression (i.e., LCMV infection) in splenic iregDC and stimDC (gated on CD39 and CD95) on day 9 after infection. In parallel, naïve splenic DC (N), and iregDC and stimDC were FACSorted based on GFP expression on day 9 after infection. The level of GFP+ stimDC was too small to obtain adequate numbers for RNA isolation. Bar graphs show relative expression of IL-10 RNA, LCMV-GP RNA and the MFI of PDL1 protein expression from the sorted populations. To obtain sufficient cell numbers for the RNA analyses, infected and uninfected iregDC and stimDC were sorted from a pool of 8 mice. Data show one of two experiments. **B.** Flow plots show the percent and bar graphs the number of iregDC and stimDC in the indicated mouse strain from CD11b+ DCs at day 9 after LCMV-Cl13 infection. **C.** Bar graphs indicate plasma IL-10 concentration and PDL1 expression in WT, IFNαR-/-, and MyD88-/- mice at day 9 after LCMV-Cl13 infection. **D.** Naïve WT and MyD88-/- monocytes were transferred into WT mice and analyzed on day 9 after LCMV-Cl13 infection as described in Fig 4E. **E.** Mixed chimera mice were generated using bone marrow from WT and MyD88-/- bone marrow. Flow plots depict WT and MyD88-/- lineage splenic CD11b+ DCs at day 9 after LCMV-Cl13 of WT. Histogram shows PDL1 expression on iregDCs from WT (white) and MyD88-/- (grey) lineage cells. Bar graphs indicate PDL1 expression and the number of iregDC and stimDC from each lineage in the chimera mice. Data are representative of 2 or more independent experiments each consisting of 3–5 mice per group. *, p<0.05.

IFN-I signaling is necessary for iregDCs to acquire their immunosuppressive capacity, yet the mechanisms that induce the development of iregDC during viral persistence remain unclear. iregDC arise during inflammation, but are not observed at appreciable levels under steady state conditions in naïve mice (Fig 1B). Direct virus infection has been proposed as a requirement for IL-10 production by DC during viral persistence [22, 23], although the exact relationship is unclear [6]. To explore whether direct virus infection induces the IFN-I production that would then drive IL-10 and PDL1 expression, we identified productively infected cells using an LCMV-Cl13 variant expressing GFP [24]. Nearly all the infected GFP+ DCs were iregDCs, but the majority of iregDCs were not infected (Fig 5A). Interestingly, the stimDC did not express GFP (Fig 5A), suggesting that monocyte-derived DCs (moDCs) and not bona-fide CD11b+ cDCs are the main DC reservoir of LCMV infection at day 9 after LCMV infection. Importantly, the predominant infection of iregDC was also observed in non-CD8 depleted WT mice stained with an anti-LCMV antibody (S3A Fig). At day 25 of infection, the frequency of virus infected DCs dropped dramatically, although CCR2+ DCs remained the major infected DC subset (S3B Fig). Further, GFP+ and GFP- iregDCs expressed similar levels of IL-10 and PDL1 regardless of infection status, while the stimDCs expressed very little of either (Fig 5A). GFP+ iregDC expressed high levels of LCMV-GP RNA, and although very low levels of LCMV RNA were observed in the GFP- iregDC population, there was no correlation with amount of IL-10 production (Fig 5A). Thus, other mechanisms besides direct infection induce the IFN-I that drives IL-10 and iregDC development.

Antibody blockade of IFNβ at the onset of LCMV-Cl13 infection enhances control of virus infection, although it was substantially less effective in controlling persistent infection than anti-IFNαR blocking antibody [25]. Consistent with this, no decrease in iregDC, PDL1, IL-10 or increase in stimDC occurred in LCMV-Cl13 infected IFNβ-/- mice (S3C Fig), indicating that IFNβ alone does not drive immunosuppression or iregDC differentiation during viral persistence and suggests in part why IFNβ antibody blockade may not be as efficient for controlling infection as blocking all IFNαR signaling.

Pathogen and damage recognition are important mechanisms associated with inflammation during persistent infection that could induce the monocyte to DC/iregDC transition. LCMV replication and the cell stress/ damage it induces are recognized by multiple toll-like receptors (TLRs) and intracellular recognition pathways [26, 27]. After being activated, most TLRs signal through MyD88 or Trif (TLR3). Further, RIG-I and MDA5 recognize intracellular LCMV as well as cellular stress/ damage through interaction with the adapter protein MAVS [26, 27]. To test the role of these pathways, we infected MAVS-/- (Cardiff-/-), MyD88-/-, and Trif-/- (TLR3 signaling deficient) mice with LCMV-Cl13. MAVS-/- mice exhibited an increased number of stimDC, without a change in the number of iregDCs or IL-10 expression (although a small decrease in PDL1 expression on iregDC in MAVS-/- mice was observed; Fig 5B and S3D Fig), suggesting that MAVS signaling is important for the suppression of stimDC during persistent virus infection, but with minimal effect on iregDC differentiation. Trif-/- mice exhibited no change in iregDC or stimDC numbers (Fig 5B), indicating that TLR3 signaling is not required for development of iregDC or suppression of stimDCs. Conversely, MyD88-/- mice exhibited a substantial loss of iregDCs numerically, without the increase in total splenic cellularity or stimDC numbers observed in IFNαR-/- and MAVS-/- mice (Fig 5B). Further, MyD88-/- mice exhibited decreased PDL1 and plasma IL-10 levels (Fig 5C). However, MyD88-/- lineage-derived cells differentiated normally into iregDC when naïve MyD88-/- monocytes were transferred into WT LCMV-Cl13 infected mice (Fig 5D) or in infected 50:50 WT:MyD88-/- mixed bone marrow chimeras (Fig 5E). Thus, the regulation of iregDC generation by MyD88 is monocyte extrinsic and suggests that MyD88-/- signaling induces other downstream factor(s) to drive the monocyte to DC transition required for iregDC differentiation.

**Fig 6:**
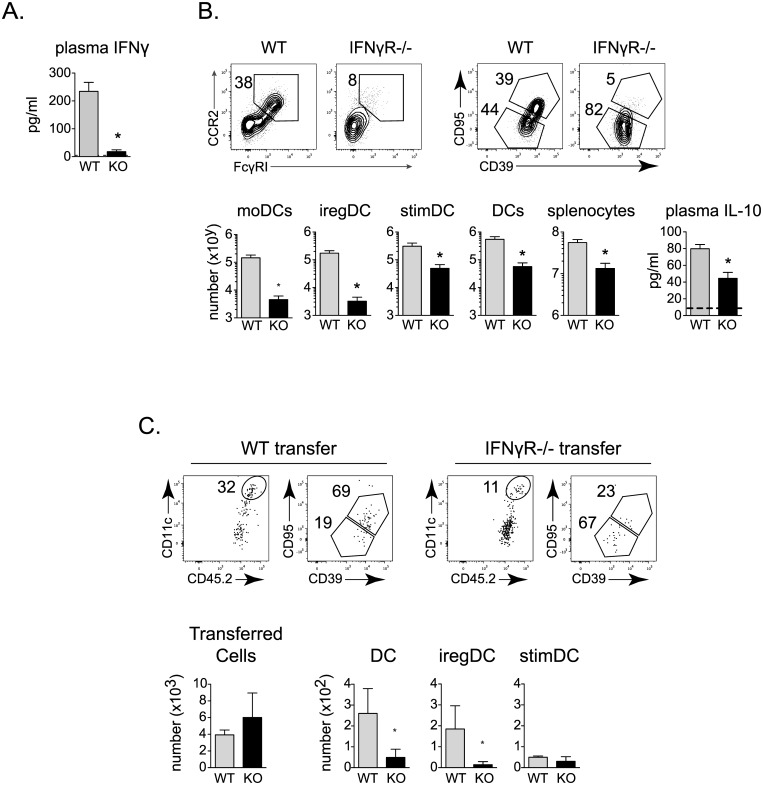
Monocytes require direct IFNγR signaling for DC differentiation throughout persistent virus infection. **A.** Plasma IFNγ concentration in WT and MyD88-/- (KO) mice at day 9 after LCMV-Cl13 infection. **B.** Flow plots represent the percent of moDC, iregDC and stimDC (gated on total CD11b+ CD11c+ DC) on day 9 in LCMV-Cl13 infected WT and IFNγR-/- mice. Bar graphs indicate the number of moDC, iregDC, stimDC, total CD11b+ DC, total splenocytes and plasma IL-10 concentration in WT and IFNγR-/- mice at day 9 after LCMV-Cl13 infection. **C.** WT and IFNγR-/- naïve monocytes were transferred into LCMV-Cl13 infected WT mice and gated and analyzed as in Fig 4E. Flow plots show CD11c++ DC, and iregDC and stimDC development in WT (left flow plots) and IFNγR-/- (right flow plots) transferred monocytes. Bar graphs indicate the total number of transferred cells at day 9 after infection and the number of CD11b+ DC, iregDC, and stimDCs derived from the transferred monocytes. Data are representative of 2 or more independent experiments each consisting of 3–5 mice per group. *, p<0.05.

IFNγ is linked to moDC development in other models [28] and plasma IFNγ levels were decreased in MyD88-/- compared to WT mice after LCMV-Cl13 infection (Fig 6A). Further, iregDCs express increased levels of the IFNγ inducible chemokines CXCL9 and CXCL10 (Fig 2), suggesting a role for IFNγ in the differentiation of iregDC. At day 9 after LCMV-Cl13 infection of IFNγR-/- mice, the number of total splenocytes was decreased ~10-fold compared to WT mice and this corresponded with a similar 10-fold decrease in the amount of total DC and stimDC (Fig 6B), suggesting that loss of IFNγ does not specifically affect stimDC maturation. On the other hand, the number of moDC decreased ~30-fold and iregDCs decreased ~60-fold in IFNγR-/- mice compared to WT mice (Fig 6B), consistent with a specific role of IFNγ signaling in moDC and iregDC differentiation. The decreased number of iregDC coincided with diminished plasma IL-10 levels (Fig 6B). Further, moDC differentiation was substantially reduced when naïve IFNγR-/- monocytes were transferred into LCMV-Cl13 infected WT mice (Fig 6C), indicating that direct IFNγ signaling is critical for the monocyte to DC transition required for IFN-I induction of immunosuppression.

### Monocyte-origin and ireg phenotype of DC expressing suppressive factors in *Mtb*, HIV and cancer

**Fig 7:**
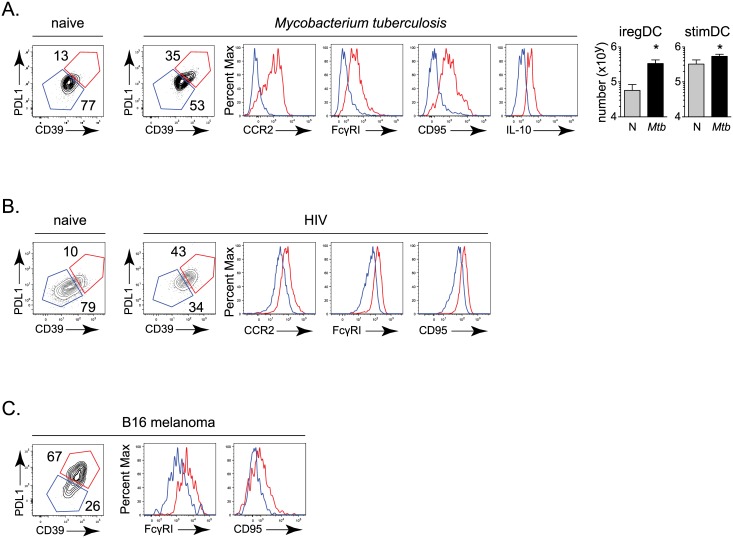
Monocyte-derived iregDCs develop in *Mtb* infection, HIV infection and cancer. **A.** Flow plots show CD39 and PDL1 expression on splenic CD11b+ DCs from IL-10 reporter mice either uninfected or infected with virulent *Mtb* for 36 days. Histograms indicate expression of the indicated protein on iregDC (red) and stimDC (blue). Bar graphs indicate the number of iregDC and stimDC in uninfected and *Mtb* infected mice. **B.** Flow plots show CD39, PDL1 and monocyte-associated proteins on CD11c++, HLA-DR+, CD14+ iregDC (red) and stimDC (blue) from the peripheral blood of naïve humanized mice and HIV infected humanized mice. **C.** Flow plots show CD39, PDL1 and monocyte-associated proteins on CD11b+, CD11c++ iregDC (red) and stimDC (blue) on B16 melanoma infiltrating leukocytes. Data are representative of 2 or more independent experiments each consisting of 4–7 mice per group. *, p<0.05.

We sought to determine whether a similar developmental origin of DCs expressing suppressive factors occurs in other chronic diseases associated with immunosuppression, inflammation, IFNγ and IFN-I signaling. *Mtb* infection is associated with both IFNγ and IFN-I signaling, with worse disease correlated with increased IFN-I and IL-10 expression [29–32]. Using a mouse model of pulmonary tuberculosis, in which mice are aerosol infected with the highly virulent Erdman strain of *Mtb*, we observed that the PDL1 and IL-10 expressing DCs also exhibited heightened expression of the monocyte markers CCR2 and FcγRI in conjunction with increased levels of the iregDC markers CD39 and CD95 by 30 days after infection (Fig 7A), suggesting that similar to persistent LCMV, the DC that the produce suppressive factors IL-10 and PDL1 are of monocyte origin. HIV infection is also associated with chronic IFN-I signaling, inflammation and increased levels of IL-10 and PDL1 that inhibit HIV-specific T cell response [5]. To determine if the DC with immunosuppressive potential are also of monocyte origin in HIV infection, we infected humanized BLT mice with HIV. Thirteen weeks after infection, we observed that the population of human peripheral blood cells that expressed the canonical human dendritic cell markers CD11c and HLA-DR (MHC II) in conjunction with PDL1 also expressed the monocyte markers FcγRI and CCR2 and iregDC markers CD39 and CD95 (Fig 7B). Similarly, many cancers are associated with inflammation and expression of immunosuppressive factors [13, 33]. To test whether similar differentiation of monocyte-derived DC expressing suppressive factors also occurs in a tumor setting, we injected mice with B16 melanoma cells. Again, the PDL1 expressing population of tumor infiltrating DC expressed proteins indicating monocyte origin (i.e., CCR2+, FcγRI+) and co-expressed iregDC markers CD39 and CD95 (Fig 7C). Thus, our data indicate that the DC expressing suppressive factors are of monocyte origin and have similar iregDC differentiation in multiple infectious and non-infectious chronic diseases characterized by inflammation and immunosuppression.

## Discussion

Many factors that suppress T cells to promote viral persistence are being identified, but their underlying mechanisms of induction, regulation and their cellular origins are unclear. In this study we (1) identify the origin of suppressive DC during persistent virus infection; (2) define the divergent mechanisms that lead to their generation and functional programming; (3) demonstrate that these process are separable events; (4) demonstrate that in the absence of IFN-I signaling, otherwise suppressive DC now exhibit robust T cell stimulatory capacity; (5) link the immunosuppressive DC observed in persistent LCMV infection to other persistent diseases; and importantly, (6) clarify many of the questions and controversies concerning suppressive DCs in persistent virus infection.

There has existed a significant lack of understanding of how immunosuppressive DC are generated during persistent virus infection. To address this issue, we identified markers to categorize suppressive DC without relying on their suppressive functions for characterization and demonstrated that specifically monocytes and not conventional DC gave rise to immunosuppressive DC during persistent virus infection. Further, differentiation of monocytes into suppressive DC required a direct two-hit mechanism. As virus titers and inflammatory signals continue to rise, namely IFNγ, monocytes differentiate into moDC. These moDC are generally considered pro-inflammatory [28], however, in the presence of continued IFN-I production during persistent infection, the inflammatory moDC are induced to express PDL1 and IL-10. In addition to their suppressive functions, iregDC also express high levels of T cell attracting and interacting molecules to draw-in, interact with and suppress T cell activity. Considering that both IFNγ and IFN-I are generally thought of as antiviral and immune stimulatory during viral infection, our data now provide a new mechanistic understanding that integrally links inflammation to balance immunosuppression during persistent virus infections.

In parallel to inducing suppressive factors, IFN-I production suppresses the generation of T cell stimulating cDC, further compounding the suppressive environment. IFN-I can limit the amount of DCs able to egress from the bone marrow [34], and our data indicate that this is through mechanisms distinct from those that lead to IFN-I mediated upregulation of IL-10 and PDL1 expression, in particular requiring MAVS-mediated signaling. The decrease in cDC is consistent with other persistent infections in which DC functionality is diminished and an immunosuppressive environment is established [35–39]. In fact, what is perceived as the diminished DC function may in part be due to a shift toward monocyte-derived iregDC that are phenotypically similar to cDC, but are suppressive instead of stimulatory. Thus, by directly driving immunosuppressive factors and an overall population inversion from stimulatory to suppressive DC, we provide mechanistic insight by which interferons and distinct pathogen recognition pathways collaborate to shift and shape the environment toward suppression during persistent infection.

Why some APCs become suppressive while others in the same environment do not has remained unclear. Both iregDC and stimDC express similar levels of IFNαR and in theory are both in the same inflammatory and IFN-I containing environment [10]. Our data now shed light on this matter by indicating that certain DC precursors can serve as targets for IFN-I mediated IL-10 and PDL1 upregulation *in vivo*. Without the precursor moDC population induced by IFNγ, IFN-I does not have the target cell available to induce IL-10 and PDL1, although IFN-I antiviral and immunostimulatory aspects would still be present. Our data can further help explain the tissue restriction of IL-10 and PDL1 expressing cells and T cell exhaustion to sites of infection or cancer induced inflammation [40–44]. In the presence of IFN-I induced by inflammation, the precursor moDC are signaled toward an immunosuppressive program, whereas in distant sites, even if moDC are present, the lack of infection or tumor growth would not induce IFN-I, or as a result, suppressive APC. MyD88 and IFNγ have also been shown to guide monocyte differentiation into DC in other inflammatory systems [28, 45, 46], suggesting the mechanisms underlying moDC differentiation are a conserved response to inflammation. Interestingly, although MyD88 was required for moDC generation during persistent LCMV infection, the requirements were indirect and MyD88 sensing of infection formed a foundation to induce expression of additional factors that then led to iregDC differentiation. Thus, multiple pathogen sensing steps are required to ultimately lead to the generation of suppressive DC and underscore the intricate dependence of ongoing inflammation and immunosuppression.

The ability of transferred monocytes to differentiate into iregDC at the beginning and during persistent infection suggests that their conversion is a continual process. The increased expression of CD95 and activated Caspases by iregDC would render them highly susceptible to apoptosis and rapid turnover. Interestingly, DCs lacking CD95 expression were recently observed to be more potent at sustaining the immune response during persistent LCMV infection [47]. Although in our studies iregDC expressed the highest amounts of CD95, the lack of CD95 on conventional DC may enable longer life of the conventional DC that are generated and thus, limit the stimDC to iregDC inversion. Together, these data suggest a mechanism to facilitate the highly tunable immunologic system to respond rapidly to changing inflammatory and pathogen environments.

Although many of the suppressive factors are known, an understanding of where and how the suppressive cells localize *in vivo* is limited. We show that instead of forming distinct foci of immunosuppression, IL-10 expressing cells are widely distributed throughout the red pulp and marginal zone of the spleen and ultimately are also observed in the B cell follicle consistent with temporally increasing IL-10 expression from T cells and B cells [6]. During persistent infection, T cells overlap with areas of high IL-10 expression in the spleen and are present throughout the red pulp and marginal zone during persistent LCMV infection (Fig 1A and [48]). Importantly, McGavern and colleagues [48] demonstrated that CD4 and CD8 T cell immune exhaustion in persistent LCMV infection maps to the red pulp/marginal zone of the spleen where these cells are engaged in prolonged contacts, again consistent with positioning them to interact with IL-10 producing cells. Loss of IL-10 specifically from CD11c expressing DC led to an overall decrease in IL-10 levels [22]. Further, our observation that the main IL-10 producing DC are of monocyte origin and express high amounts of LysM (Lyz2; Fig 4) may suggest an underestimate of the contribution from IL-10 expressing DC in studies that use myeloid specific promoters (LysM-Cre) to delete IL-10 [49]. Considering the breadth of cell types that are able to produce IL-10 in response to persistent LCMV infection, it is likely that each work in their own microenvironments and in concert to contribute to the IL-10 induced immunosuppressive environment. Ultimately, it will be interesting to tease apart the individual contribution of each toward establishing and sustaining the immunosuppressive environment.

There has been controversy over whether direct productive infection of the DC is required to generate its IL-10 production [6, 22, 23]. Through multiple techniques, we demonstrate that IL-10, PDL1 and iregDC generation are not specifically dependent on direct infection or recognition of intracellular viral replication by the LCMV sensors TLR3 or MAVS. Although only a fraction of iregDCs were infected, it was interesting that iregDC (i.e., moDC) are the main source of DC infection, with minimal infection of conventional CD11b+ DC. Thus, although IL-10/PDL1 producing iregDC are the main DC subset infected, their infection is not required for induction of the immunosuppressive program. It is possible that the low levels of LCMV RNA present in the GFP- iregDC are sufficient to induce robust levels of IL-10 expression. However, in the absence of IFN-I signaling the amount of IL-10 RNA drops dramatically in DC (Fig 3B), whereas the amount of infected DC increases [10], which is not consistent with direct infection triggering IL-10 production. It is further conceivable that direct infection drives the IFN-I that then is restricted to function through an autocrine fashion to increase IL-10. However, then it would be expected that only infected GFP+ cells would produce IL-10 and that IL-10 levels would directly correlate to a cells level of infection, which is not the case. On the other hand, MAVS-/- mice exhibit an increased amount of stimDC, similar to IFNαR-/- mice, suggesting that intracellular, MAVS recognition of LCMV replication is a critical step in the IFN-I mediated suppression of stimDC. Together, these data indicate that induction of IFN-I signaling orchestrates multiple aspects of immunosuppression through distinct mechanisms during persistent infection.

The identification of multiple markers that effectively differentiate IL-10/PDL1 producing suppressive iregDC from their stimulatory DC counterparts enabled analysis of their origin and generation in multiple chronic inflammatory diseases. It is important to note that PDL1 in combination with these other markers is a reasonable approach to identify iregDC in situations where IL-10 reporter mice cannot be used, and the use of PDL1 as an identifier of iregDC was only unusable when investigating the mechanisms that regulate PDL1 expression itself. Monocyte-derived iregDC phenotype cells specifically expressing suppressive factors were observed in *Mtb* and HIV infection, and cancer, suggesting that suppressive DC arise from a monocyte origin in multiple situations of chronic disease characterized by continually high antigen loads, inflammation (i.e., IFNγ, IFN-I) and immunosuppression mediated by IL-10 and PDL1 [5, 39]. Importantly, the monocyte origin of suppressive DC was also observed in human cells using a humanized mouse model of HIV infection, suggesting that similar cellular ancestries and mechanisms of induction can occur in human persistent virus infections. Ultimately, targeting these underlying mechanisms of chronic inflammation may provide a potent therapeutic strategy to simultaneously and specifically dampen immunosuppression at its foundation to bolster T cell responses to control multiple chronic infections.

## Materials and Methods

### Ethics statement

This study was performed in accordance with the recommendations in the Guide for the Care and Use of Laboratory Animals of the National Institutes of Health, and local policies in the review and approval of animal care and use protocols. The protocols were approved by the Chancellor’s Animal Research Committee (ARC) of the University of California, Los Angeles (Assurance Number: A3196-01)

### Mice and infections

C57BL/6 (wild type) mice were purchased from The Jackson Laboratory (stock# 000664) or from the rodent breeding colony at UCLA. 10BiT (IL-10/Thy1.1 reporter mice) [18] were provided by Dr. Casey Weaver (University of Alabama, Birmingham) via Dr. Gislaine Martins (Cedars-Sinai Medical Center, Los Angeles, CA) and were described previously. LCMV-GP61-80-speicifc CD4+ TCR transgenic (SMARTA) mice have been described previously [50]. *Ifnar-/-* (IFNαR-/-) mice were provided by Dr. Dorian McGavern (NINDS/NIH). *TRIFLps2/Lps2* (Trif-/-) mutant and MAVS-/- mice were provided by Dr. Genhong Cheng (UCLA). *Ifngr1* ^*tm1Agt*^/J (IFNγR-/-, stock# 003288), *Myd88* ^*tm1*.*1Defr*^/J (MyD88-/-, stock# 009088), *Ptprc* ^*a*^ *Pepc* ^*b*^/BoyJ (CD45.1, stock# 002014) and BALB/cJ (stock# 000651) were purchased from the Jackson Laboratory.

For LCMV studies, mice were infected intravenously (i.v.) via the retro-orbital sinus with 2x10^6^ plaque forming units (PFU) of LCMV-Cl13. Virus stocks were prepared and viral titers were quantified as described previously[51]. For LCMV-Cl13-GFP (provided by Juan Carlos de la Torre, The Scripps Research Institute) studies, mice were treated with 125μg of anti-CD8 depleting antibody (BioXcell—clone 2.43) i.v. via the retro-orbital sinus 5 and 2 days before being infected with 8x10^4^ PFU of LCMV-Cl13-GFP.

For *Mtb* studies, mice were infected with 100 colony forming units (CFU) of *Mtb* Erdman strain via exposure for 30 minutes within an aerosol chamber to an aerosol generated by a Collison Type 6-jet nebulizer (BGI, Inc.) at 20 psi from a suspension containing 7.5x10^5^ CFU/mL *Mtb* in PBS. The precise number of bacteria used in the aerosol was determined by plating serial dilutions of the infection stock on Middlebrook 7H11 agar plates and enumerating bacterial CFU. One day after infection, two mice were euthanized to determine the initial number of bacteria delivered to the lungs.

For HIV studies, BLT mice were constructed similarly to what was reported previously [52]. Briefly, CD34+ cells were purified via magnetic activated cell sorting with CD34 specific beads (Miltenyi) from freshly obtained fetal liver tissue. NSG mice were transplanted with fetal liver stromal and fetal thymus derived from the same tissue. Before surgery, the transplant mice were sublethally irradiated (3Gy) and 5x10^5^ transduced CD34+ cell were injected into the each mouse after surgery. Six to 12 weeks later, peripheral blood was collected and analyzed for human cell reconstitution; typically 40–90% of the peripheral blood cells are human cells. Following confirmation of reconstitution, the mice were then infected with HIV-1NL4-3 (300ng p24, as determined by ELISA) by intraperitoneal (i.p.) injection. Human fetal tissue to produce humanized mice was purchased from Advanced Biosciences Resources or from Novogenix Laboratories and was obtained without identifying information and did not require IRB approval for its use. Specifically, these studies were carried out under strict accordance to the guidelines in The Guide for the Care and Use of Laboratory Animals of the National Institutes of Health and the accreditation and guidelines of the Association for the Assessment and Accreditation of Laboratory Animal Care (AALAC) International. All surgeries were performed under ketamine/xylazine and isofluorane anesthesia and all efforts were made to minimize animal pain and discomfort.

For tumor studies, mice were subcutaneously injected with 1x10^6^ B16 melanoma cells in close proximity to the inguinal lymph nodes.

### Flow cytometry

Analysis of mouse immune cells was performed by staining directly *ex vivo* for viability using Zombie Aqua fixable viability dye (Biolegend). Dendritic cells (whether cDC or monocyte-derived) were defined as: viable (based on viability dye exclusion), CD45+, Thy1.2-, NK1.1-, Ly6G-, CD11c++ (high), CD11b+. Cells were subsequently stained for intra- and extra-cellular analysis using the following antibodies purchased from Biolegend and eBioscience (except where noted): CD45.1 (clone A20), CD45.2 (clone 104), or CD45 (clone 30-F11), CD90.2 (clone 30-H12), CD3ε (clone 145-2C11), NK1.1 (clone PK136), Ly6G (clone 1A8), CD11c (clone N418), CD11b (M1/70), F4/80 (clone BM8), PDL1 (clone 10F.9G2 and clone MIH5), CD95 (clone 15A7), CD39 (clone 24DMS1), CD90.1 (clone HIS51), MHC I (H-2K^b^/H-2D^b^; clone 28-8-6), MHC II (I-A/I-E; clone M5/114.15.2), CD80 (clone 16-10A1), CD86 (clone GL-1), CCR2 (R&D Systems; clone 475301), FcγRI (clone X54-5/7.1), FcεRIα (clone MAR-1), FcγRII/III (clone 93), Ly6C (clone HK1.4), CD172α (clone P84), B220 (clone RA3-6B2), CD4 (clone GK1.5), and CD8 (clone 5.3–6.7) and CD68 (clone FA-11). LCMV infection was measured by intracellular staining with anti-LCMV nucleoprotein (Bioxcell; clone VL-4) conjugated to Alexa Fluor 647 (Molecular Probes). Pan-Caspase activity was assayed directly *ex vivo* by staining with FAM-FLICA Poly Caspase Assay (ImmunoChemistry Technologies). For all Figures, flow plots shown represent the average (i.e., one of the middle values) of the group.

In studies with *Mtb* infected splenocytes, cells were fixed in 4% formaldehyde in PBS for 1h at room temperature to inactivate *Mtb* prior to flow cytometric analysis. Human cells within the BLT mouse were stained directly *ex vivo* for the expression of huCD45 (clone HI30), huCD3 (clone OKT3), huCD337 (clone p30-15), huCD19 (clone HIB19), huHLA-DR (clone LN3), huCD14 (Beckman Coulter; clone RMO52), huCD16 (BD Bioscience; clone 3G8), huCD11c (clone 3.9), huPDL1 (BD Bioscience; clone MIH1), huCD95 (clone DX2), huCD39 (clone A1), huCCR2 (clone K036C2), and huFcγRI (clone 10.1). Flow cytometric analysis was performed using a FACSVerse (BD) or LSRFortessa (BD). Data was analyzed using FlowJo version 9 and 10 (Treestar).

### Purification of APC subsets

For iregDC and stimDC sorting experiments, mouse splenocytes were pooled and enriched for CD11c positive cells by magnet activated cell sorting with CD11c MicroBeads (Miltenyi). Enriched cells were then extracellularly stained for iregDCs and stimDCs and sorted on a FACSAria III (BD). For LCMV-Cl13-GFP experiments each mouse was tested for GFP+ (infected) cells before being pooled, enriched, stained for iregDCs and stimDCs and sorted on a FACSAriaIII (BD). For naïve monocyte sorting experiments bone marrow cells were pooled, enriched using a monocyte negative selection kit (StemCell Technologies), stained for monocytes (CD45+, CD90.2-, NK1.1-, Ly6G-, CD11b+, CCR2+, and Ly6C+), and sorted on a FACSAriaIII (BD). For naïve conventional DC sorting experiments splenocytes were pooled, enriched for CD11c positive cells using CD11c microbeads and an autoMACS (Miltenyi), and sorted on a FACSAriaIII (BD). Post sort purity was >98%.

For experiments in which intrahepatic DC were analyzed, the mice were perfused with 25–30 ml of 0.9% saline by direct cardiac injection to remove blood from all tissue compartments. Intrahepatic lymphocytes were further isolated by centrifugation in 35% Percoll.

Tumor infiltrating leukocytes were harvested 2 weeks after tumor injection. Tumors were digested in a solution containing 0.5mg/mL Collagenase D (Sigma Aldrich) for 60 minutes at 37°C with frequent agitation and resuspension. Digested samples were then passed through a 100μm filter, resuspended in a solution of 30% Percoll (GE Healthcare), and laid over a solution of 70% Percoll. After centrifugation, the tumor infiltrating lymphocytes and leukocytes were harvested from the interface between the 30% and 70% Percoll fractions.

### *In vitro* T cell stimulation

Antigen specific SMARTA CD4+ T cells were isolated from the spleens of naïve SMARTA mice and purified by negative selection (StemCell). T cells were labeled with 2.5 μM CFSE (Invitrogen) and cultured for 3 days with sorted iregDCs or stimDCs at a ratio of 2:1 T cells to dendritic cell populations (40,000 T cells to 20,000 iregDCs or stimDCs). Antibodies against PDL1 (BioXcell; clone 1F.9G2) or IL-10R (BioXcell; clone 1B1.3A) were added at a concentration of 10 μg/ml. Percent proliferation was measured by CFSE dilution and quantified as in Gett et al. [21].

### Monocyte transfer

Bone marrow was harvested from naïve donors and enriched for monocytes using a monocyte negative selection kit (StemCell Technologies). Enriched monocytes were injected i.v. via the retro-orbital sinus of mice infected with LCMV-Cl13 on day 3 of infection. Spleens were harvested at day 9 post infection and analyzed by flow cytometry. The number of monocytes transferred varied between experiments (between 2.5x10^6^ and 6x10^6^ million cells) but was held constant among groups within experiments.

### Bone marrow chimeras

Naïve mice were lethally irradiated with 950 rads and subsequently given a 50:50 mixture of bone marrow harvested from naïve WT and KO donors. Mice were given a total of 2x10^7^ bone marrow cells i.v. via the retro-orbital sinus and treated for 4 weeks with antibiotics (Sulfamethoxazole and Trimethoprim in the drinking water) to prevent infection and allow for immune reconstitution. Reconstitution was quantified 8 weeks post transfer and the mice were subsequently infected.

### ELISA

Plasma samples were assayed for IL-10 and IFNγ by ELISA per the manufacturer’s instructions (R&D Systems).

### Quantitative Real Time Polymerase Chain Reaction (qRT-PCR)

RNA was harvested from sorted cell populations and isolated with the RNeasy Extraction Kit (Qiagen). cDNA was generated from the isolated RNA using iScript cDNA synthesis kit (BioRad). The expression of *IL-10*, *CXCL9*, and *CXCL10* were normalized to *HPRT*. *IL-10*, *CXCL9*, *CXCL10*, and *HPRT* were amplified using Assays-on-Demand TaqMan premade primer probes (Applied Biosystems) using an iCycler iQ (Bio-Rad).

### Microscopy

Fresh spleens were embedded in optimal cutting temperature (OCT; TissueTek) and frozen on dry ice. After cryostat sectioning at 6-μm, spleens were fixed with 4% PFA for 20 minutes and treated with 3% Hydrogen Peroxide for 30 minutes before blocking with an Avidin/Biotin Vector Labs Blocking Kit and 2% Fetal Bovine Serum. Primary antibody incubation included CD4-FITC (BD Biosciences), B220-APC (Biolegend), and Thy1.1-Biotin (Biolegend; clone OX-7). After primary antibody incubation, sections were washed in PBS before secondary amplification was performed using streptavidin (SA)-Rhodamine Red-X (Jackson Immuno Research Labs). For additional rounds of secondary amplification, sections were washed with PBS before incubation with biotinylated anti-SA (Vector Labs), washed, and stained again with SA-Rhodamine Red-X. The amplification process was repeated once more for a total of 3 amplifications with Rhodamine Red-X. Sections were visualized using the Zeiss Axio Observe Z1 immunofluorescence microscope (Carl Zeiss, Inc.) and images were taken using an AxioCam MRm camera with a 10x objective (Carl Zeiss, Inc.). Images were prepared using Adobe Photoshop.

### RNA-Seq

iregDCs and stimDCs (spleen) were FACSorted based on CD39 and CD95 expression 9 days after LCMV-Cl13 infection. In parallel, naive monocytes (bone marrow) and cDCs (spleen) were FACSorted from naïve mice. Total RNA from each population was harvested using the RNeasy extraction kit (Qiagen), converted into double-stranded cDNA and amplified using the Ovation RNA-Seq System V2 (NuGEN Technologies). Sequencing libraries were then generated from the amplified cDNA product using the Kappa LTP library preparation kit (Kappa Biosystems). Briefly, the workflow included fragmentation of double stranded cDNA, end repair to generate blunt ends, A-tailing, adaptor ligation and PCR amplification steps. Sequencing was performed on an Illumina NextSeq 500 sequencer to obtain between 41–52 million uniquely mapped 75bp single-end reads per sample. Data quality check was done on Illumina Sequencing Analysis Viewer (SAV). Demultiplexing was performed with Illumina CASAVA 1.8.2. Reads were mapped onto mouse genome build GRCm38/mm10 using TopHat [TopHat v2.0.13, http://tophat.cbcb.umd.edu [53]] with settings ‘—library-type fr-unstranded—no-novel-juncs’. HTSeq [v0.6.1, http://www.huber.embl.de/users/anders/HTSeq/doc/overview.html [54]] was used to process read alignments produced by TopHat. Counts for uniquely mapped reads were obtained for each gene across all samples using htseq-count and with the gene model file genes.gtf associated with the Illumina iGenomes UCSC (mm10) release. Total number of uniquely mapped reads and gene length data were used to calculate RPKM expression values and all genes with a value of 2 RPKM or more in at least one sample were considered for further analysis. The gene expression data is deposited in the Gene Expression Omnibus (GEO), Accession number GSE75767, URL: http://www.ncbi.nlm.nih.gov/geo/query/acc.cgi?acc=GSE75767.

For gene ontology (GO) analysis, genes that were differentially expressed 3-fold or higher in the indicated pairwise comparisons were selected. The online Functional Interpretation of Differential Expression Analysis tool [FIDEA, http://circe.med.uniroma1.it/fidea/ [55]] was used to annotate gene lists with GO terms associated with the Biological Process (BP) category.

RPKM values for all genes expressed above the 2 RPKM cutoff were used to perform hierarchical clustering using the R ‘stats’ package [(R Core Team (2013). R: A language and environment for statistical computing. R Foundation for Statistical Computing, Vienna, Austria. ISBN 3-900051-07-0)]. A Euclidean distance matrix was calculated using the ‘dist’ function and used to plot a dendrogram with the ‘hclust’ function using the ‘ward.D2’ method. Log-transformed RPKM values for iregDC and stimDC samples were plotted using the R package ‘ggplot2’.

### Statistics

Student’s t tests (two-tailed, unpaired) were performed using GraphPad Prism 5 software (GraphPad Software, Inc.).

## Supporting Information

S1 Fig *In vivo* IL-10 microscopy controls and iregDC differentiation in BALB/c mice. **A. S**ections from LCMV-Cl13 infected WT (i.e., non-IL-10 reporter) mice were stained for B cells, CD4+ T cells, and CD90.1 on the indicated day. Sections were visualized at 10x magnification. **B.** Flow plots demonstrate the gating strategy used to identify dendritic cells. The Dump includes antibodies against NK cells (anti-NK1.1), T cells (anti-Thy1.2) and neutrophils (Ly6G). **C.** Similar to Fig 1B. CD39 and CD95 expression on liver-infiltrating, inguinal and mesenteric lymph node CD11b+ DCs from naive IL-10 reporter mice or IL-10 reporter mice infected with LCMV-Cl13 for 9 days and their corresponding expression of CD90.1 (IL-10) and PDL1 within the iregDC (red) and stimDC (blue) populations. Bar graphs indicate the geometric mean fluorescence intensity (MFI) of CD90.1 (IL-10) and PDL1 expression by iregDCs (red) and stimDCs (blue). **D.** Flow plots and bar graphs illustrate iregDC and stimDC differentiation in C57BL/6J and Balb/cJ mice on day 9 after LCMV-Cl13 infection. Data are representative of 2 independent experiments consisting of 4 mice per group. *, p<0.05.
(EPS)

S2 Fig Reconstitution of the WT: IFNαR-/- mixed bone marrow chimera mice pre- / post- LCMV-Cl13 infection and pre-transfer naïve monocyte phenotype. **A.** Flow plots and bar graphs show the reconstitution of WT (CD45.1+) and IFNαR-/- (CD45.2+) lineage cells in bone marrow chimera mice pre-infection (blood– 9 weeks post reconstitution) and post-infection (spleen—day 9 of LCMV-Cl13 infection). **B.** (Left flow plots) Phenotype of naïve monocytes isolated from the bone marrow of WT mice prior to transfer into LCMV-Cl13 infected mice (see Fig 4E). Data are representative of 2 or more independent experiments consisting of 3–5 mice per group.
(EPS)

S3 Fig Neither direct infection nor IFNβ are required to generate iregDCs. **A.** iregDC and stimDC from WT (non-CD8 depleted) mice stained for LCMV-Nucleoprotein expression on day 9 after LCMV-Cl13 infection. **B**. Flow plots illustrate LCMV infection on splenic CD11b+, CD11c++ DC on day 25 after LCMV-Cl13 infection in WT mice. **C.** Flow plots show iregDC and stimDC differentiation in WT and IFNβ-/- mice in the spleen on day 9 after LCMV-Cl13 infection. Bar graphs indicate the number of iregDC and stimDC, the MFI of PDL1 expression on iregDC and the level of plasma IL-10 on day 9 after LCMV-Cl13 infection. **D.** Graphs indicate plasma IL-10 levels and flow cytometric MFI of PDL1 expression in the indicated pathogen recognition receptor deficient mice. Data are representative of 2 independent experiments consisting of 3–5 mice per group. *, p<0.05.
(EPS)
